# Traffic signal active control method for short-distance intersections

**DOI:** 10.1371/journal.pone.0319804

**Published:** 2025-03-14

**Authors:** Yulin Tian, Shuqing Liu, Lu Wei, Zhen Li, Shaohu Tang, Yuchen Zhang, Tao Zhu

**Affiliations:** 1 School of Traffic and Transportation, Beijing Jiaotong University, Beijing, China; 2 Beijing E-Hualu Information Technology Co., Ltd., Beijing, China; 3 College of Urban Safety Engineering, Beijing Polytechnic College, Beijing, China; 4 China Software Testing Center, Beijing, China; 5 College of Urban Rail Transit and Logistics, Beijing Union University, Beijing, China; 6 Beijing General Municipal Engineering Design & Research Institute Co., Ltd., Beijing, China; University of Shanghai for Science and Technology, CHINA

## Abstract

Aiming at the existing problems about the overflow prevention goal and the overall traffic efficiency guarantee being difficult to optimize at the same time in the signal control process of short-distance intersections scenario, this paper proposes a traffic signal active control method based on key state prediction. In order to construct the key state evolution trend of short-distance intersection scenarios, this paper proposes the concept of overflow index for short-distance road sections and designs the prediction method of overflow index. In order to perform fast computation and solution for the active control scheme, this paper builds a solution algorithm based on deep reinforcement learning and optimizes the problem of reward sparsity in the algorithm, which improves the ability of active control in terms of state space and reward function. The experimental results show that this method can not only ensure the overall traffic efficiency of short-distance intersections and reduce the travel delay but also can actively sense the change of overflow state, improve the overflow prevention and control ability of the target scenario, and reduce the overflow risk.

## Introduction

As the total number of vehicles in cities continues to rise, problems such as traffic congestion continue to increase. When the demand for traffic flow on the road exceeds the road’s actual capacity, the oversaturated traffic phenomenon occurs, and it starts from some scenarios that are sensitive to the saturation of traffic flow. Short-distance intersections are the most typical traffic scenarios, which are a type of intersection with a short distance, but with large traffic flow and queuing overflow phenomena that easily occur.

Short-distance intersection congestion is a key problem of urban road traffic, especially in the peak hours of large travel volume, which seriously affects the travel efficiency of residents, but also causes a series of problems such as traffic safety and air pollution. Therefore, it is particularly important to carry out the research on the targeted control methods for the congestion problem of short-distance intersections.

Michalopoulos et al. [1] designed a control strategy that considers the queue length of short-distance road sections, which takes the overall delay of the intersection as the goal and carries out a detailed design and validation of the control model. Since the method takes the overall efficiency as the key index, it can ensure the overall operation effect of upstream and downstream intersections, but due to the fact that it ignores the role of the key roadway queue length index, the method is not effective in practical use; it cannot effectively avoid the occurrence of overflow. Lu et al. [2] modeled the capacity of short-distance intersections and the correlation between intersections, which helped to analyze the factors affecting the efficiency of short-distance intersections, but did not give specific signal control strategies and methods. Yang et al. [3], Liu et al. [4], and Qi et al. [5] designed a signal optimization method based on the relationship between phase difference and traffic release to achieve the prevention and control of overflow by reducing the stopping number of vehicles, which has a certain effect on the reduction of the overflow risk, but lacks the consideration of the overall traffic efficiency index of the intersection. Liu et al. [6], Li et al. [7] and Wu et al. [8] designed the overflow prevention and control method considering the intersection passing efficiency and queue length constraints, and the experimental results show that it has been effective. Zhu et al. [9] relatively complete analysis of the correlation factors affecting the capacity of short-distance scenarios; the content of this research has a certain role in guiding subsequent scholars to carry out research in the traffic flow prediction and overflow control of short-distance intersections [10–13].

Researchers’ studies on active control of traffic signals can be categorized into macro traffic and micro traffic. Research in the macro aspect mainly focuses on the state of the road network. Zhang et al. [14] proposed a hierarchical boundary control method around oversaturated intersections to alleviate the traffic pressure at intersections based on the idea of active control of key areas in the road network; Ma et al. [15] established correlation coefficients between intersections in an oversaturated traffic environment by analyzing the distribution of traffic flow at key intersections that affects the effect of regional traffic control, and then applied them to the dynamic delineation of the traffic sub-area and realized the optimal control of oversaturated road network subareas. Some researchers [16–18] have proposed new approaches to traffic signal optimization based on connected vehicle data and fuzzy control, which provide new perspectives for research in congestion control. These studies take the total amount of traffic in the road network and the overall capacity of the road network as system inputs, and solve the control strategy by modeling the matching degree of traffic supply and demand to generate control actions for regional traffic, including restricting the traffic flow into and increasing the traffic flow out of the target road network, as a way to achieve active regulation of intersections within the road network. Research in the microscopic aspect mainly focuses on the traffic flow at intersections. The active control method based on traffic flow prediction [19–24] can fully explore the traffic flow state information, more accurately describe the traffic demand in different time periods, and provide an important and accurate reference for the generation of optimal actions of real-time traffic signals. Due to the integration of traffic state prediction, this active control method can effectively improve the operation efficiency of road network traffic flow based on obtaining the direction of traffic evolution, and regulate the evolution trend of oversaturated traffic in a timely manner, which is worthy of further research.

Most of the researches take single objectives such as main-direction travel efficiency and overflow control as the control objectives, and lack designing optimization methods from the perspective of the upstream and downstream intersections as a whole. On the other hand, most of these studies are real-time control methods, which generate control schemes based on the real-time state and their passive adaptation to changes in traffic demand. The process responds to the traffic demand with a lag, resulting in ineffective control strategies. And there is a lack of state prediction of the influencing factors of the key indicators (overflow prevention and control indicator), and there is no research on the signal control of this scenario from a relatively proactive direction.

Based on the above research, in order to realize the overflow prevention and control objectives of short-distance intersections and the improvement of the overall traffic efficiency of upstream and downstream intersections, this paper proposes an active control method based on the prediction of short-duration overflow in the target road section and the deep reinforcement learning algorithm. By modeling and predicting the overflow state of the target road section, the evolution trend of the traffic state is perceived. By constructing a reinforcement learning model, the real-time state, evolution trend, and multi-dimensional indicators of the control objective are fused and solved to realize the dual control objectives of overflow prevention and control and overall traffic efficiency.

## Methods

Considering that the target scenario studied in this paper requires coordinated control of two intersections associated with a short-distance road section, the control scenario involves not only the optimization of the travel efficiency of the two intersections, but also the impact of road section overflow. Facing this multi-input coordinated optimization problem, this paper designs a reinforcement learning algorithm to model and solve this multi-input and multi-constraint problem.

Reinforcement learning algorithm through the “state-action-reward” mechanism, and constantly accumulate experience. However, since the state data of the short-distance intersections scenario can only directly reflect the queuing information of upstream and downstream intersections in each direction, the information related to overflow needs to be acquired slowly by the algorithm through deep learning, and it needs to appear repeatedly in the state of overflow in order to optimize the learning parameters continuously. This kind of passive control method, which learns from experience through the occurrence of “mistakes”, greatly reduces the application of learning algorithms in this traffic scenario.

**Fig 1:**
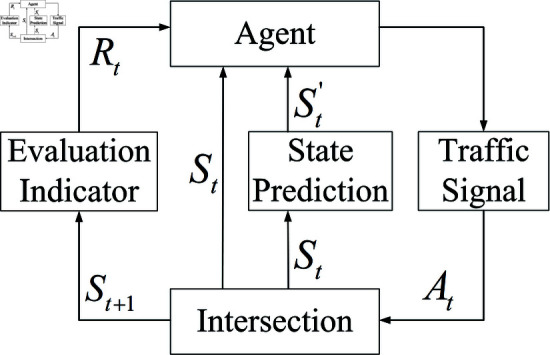
Reinforcement learning process based on overflow prediction.

In order to make up for the deficiency of the reinforcement learning algorithm in the “learning ability of the overflow process,” this paper evaluates the potential overflow risk of the short-distance road section according to the state of the traffic signal and the state of the vehicle distribution, and establishes the overflow prediction index of the target road section. This paper describes and predicts the parameter index of “overflow possibility of road section” under the current state, and then improves the cognitive ability of the reinforcement learning algorithm on “overflow generating process,” and realizes the active control of the short-distance road section based on the short-term overflow prediction. This control process is shown in Fig 1, in which the traffic states at the intersection are the algorithm inputs, and these states include the distribution states of vehicles and the states of the traffic signaling scheme. In this algorithm, the control system (Agent) collects the real-time traffic state data ($S_{t}$) of the intersection and the predicted state data ($S_{t}^{\prime }$) output from the prediction module (State Prediction), and generates the signal control action ($A_{t}$) of the intersection based on the signal action evaluation ($R_{t}$) output from the evaluation module (Evaluation Indicator), and generates the better system control parameters through continuous learning to realize the active control process.

### Overflow index model

**Fig 2:**
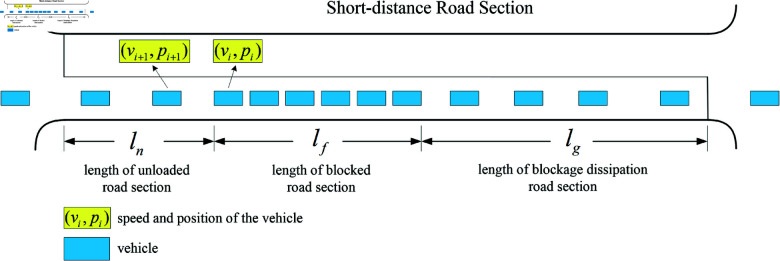
Relevant definitions in short-distance road section.

The two key factors for the occurrence of overflow in a road section are the length of queuing traffic in the road section (length of blocked road section) and the length of the upstream section of the road section that is not occupied by queuing traffic (length of unloaded road section). In this paper, the Overflow Index (IO) is defined to consist of two parts (Fig 2), namely, the ratio of the remaining roadway length to the total length of the target roadway section (overflow parameter, OP) and the ratio of the blocking roadway length to the total length of the target roadway section (blocking parameter, BP), which together portray the specific process of roadway overflow generation.

The specific form of the Overflow Index is as follows:


$$\begin{array}{l} IO=(OP,BP) \end{array}$$ (1)

The specific form of the *OP* and *BP* are as follows:


$$\begin{array}{l} OP=\frac{l_{n}}{L},BP=\frac{l_{f}}{L},l_{n}\leq L,l_{f}\leq L \end{array}$$ (2)

The *BP* in this overflow index describes the length of the road section occupied by the blocking traffic, which focuses on the dissipation process of the blocking convoys, while the *OP* focuses on the accumulation process of the blocking convoys, and the combination of the two is able to depict the process of generating and evolving the overflow well.

### Traffic flow arrival model

The traffic flow driving into the short-distance section mainly consists of left turn, straight, and right turn in the exit lane of the upstream intersection, and the arrival rate of the traffic flow exiting from the exit of the upstream intersection is modeled here. Since right-turning vehicles are not controlled by signals, their arrival rate can be approximated as a constant traffic flow arrival rate without changing the turning flow ratio, then the upstream intersection exit arrival rate $S_{a,\text{ out }}^{r}(t)$ at time *t* is


$$\begin{array}{l} S_{a,\text{ out }}^{r}(t)=\frac{q_{a,b}^{r}\times \vartheta_{a,b}^{r}}{3600} \end{array}$$ (3)

where $q_{a,b}^{r}$ is the traffic flow in the direction of the right-turn connection between upstream intersection *a* and intersection *b*, and $\vartheta_{a,b}^{r}$ is the flow proportionality factor for right-turning traffic in that direction.

Since the straight and left-turn traffic flows at the upstream intersection are influenced by signals when approaching the short-distance section, the traffic flow movement process is modeled here in two time periods: the initial green light and the end of the green light. Vehicles exit the intersection at the saturation flow rate during the initial green light period, and vehicles exit the intersection at the arrival rate of the entry lane during the end green light period. Therefore, the arrival rate of traffic flow at the upstream intersection for straight and left turns into the short distance section can be calculated as follows:


$$\begin{array}{l} S_{a,\text{ out }}^{\text{str}}(t)=\{\begin{array}{c} OS_{a,b}^{\text{str}},t\in G_{a,b}^{\text{str, start}}\\ \frac{q_{a,b}^{\text{str}}\times \vartheta_{a,b}^{\text{str}}}{3600},t\in G_{a,b}^{\text{str},\text{end }}\\ 0,t\in \text{ else } \end{array} \end{array}$$ (4)

$$\begin{array}{l} S_{a,\text{out}}^{\text{left}}(t)=\{\begin{array}{c} OS_{a,b}^{\text{left}},t\in G_{a,b}^{\text{left},\text{start}}\\ \frac{q_{a,b}^{\text{left}}\times \vartheta_{a,b}^{\text{left}}}{3600},t\in G_{a,b}^{\text{left},\text{end}}\\ 0,t\in \text{ else } \end{array} \end{array}$$ (5)

where $S_{a,\text{out}}^{\text{str}}(t)$ and$S_{a,\text{out}}^{\text{left}}(t)$ are thearrival rates of traffic flows going straight and turning left from upstream intersection*a*, $OS_{a,b}^{\text{str}}$and $OS_{a,b}^{\text{left}}$are the saturation flow rates of traffic flows going straight andturning left from upstream intersection *a* into intersection *b*,$q_{a,b}^{\text{str}}$ and$q_{a,b}^{\text{left}}$ are thetraffic flows going straight and turning left in both directions at upstream intersection *a*,$\vartheta_{a,b}^{\text{str}}$ and$\vartheta_{a,b}^{\text{left}}$ arethe proportions of the corresponding straight and left traffic flows to the total flow,$G_{a,b}^{\text{str},\text{start}}$,$G_{a,b}^{\text{str},\text{end}}$ and$G_{a,b}^{\text{left},\text{start}}$,$G_{a,b}^{\text{left},\text{end}}$ arethe green light start moments and green light end moments corresponding to thetraffic flow on the short-distance road section, respectively.

### Overflow index prediction model

The definition of overflow index focuses on the static description of the distribution of traffic flow and overflow state on the road section, while in the actual traffic scene, the distribution of traffic flow may be changing from time to time, and the overflow index is also in a time-varying state, and the short-term prediction of which can make the formulation of the control scheme more proactive. The prediction of overflow index is to analyze the conditions affecting the change of traffic flow on the road section, establish a time-varying model of traffic flow to match, and then realize the short-term prediction of the overflow process. In order to quickly build a prediction model with scenario characteristics, it is assumed here that the traffic flow in the short-distance road section is released in the form of straight and left-turn at the same time, and the straight right is a shared lane.

According to the schematic diagram of vehicle queuing in a short-distance road section, the length of the blocked road section $l_{f}$ and the length of the unloaded road section $l_{n}$ need to be solved first before the prediction of the overflow index. According to the speed characteristics of vehicle queuing in the road section, $l_{f}$ and *l*_*n*_ can be solved as follows:


$$\begin{array}{l} l_{f}=\max\left(P_{i}-P_{j}\right),\forall v_{x}\leq V_{ss},i\leq x\leq j \end{array}$$ (6)

$$\begin{array}{l} l_{n}=\min\left(P_{\text{max}}-P_{i}\right),\forall v_{x}\leq V_{ss},i\leq x\leq j \end{array}$$ (7)

where *i* , *j*is the ID code of the vehicle in the roadway,$v_{x}$ is the speed of thevehicle numbered *x*, $V_{ss}$is the speed judgment threshold of the blocking convoy; when$v_{x}\leq V_{SS}$,it means that the vehicle is in a parking queuing state, and when$v_{x}>V_{SS}$it means that the vehicle is in a movement state,$P_{i}$ is the end position ofthe vehicle queuing, $P_{j}$is the start position of the vehicle queuing, and$P_{i}-P_{j}$is the length of the roadway section occupied by the queuing vehicle, and$P_{\text{max}}$ is thecoordinates of the end of the roadway section.

Since the state of the signal lights at the entrances and exits of the short-distance road section affects the change of traffic flow in the target road section, the change state of the overflow index under different signal lights is modeled here. For the convenience of description, the upstream entrance traffic lights are defined here to control the left-turning or straight-traveling vehicles, respectively; and the downstream exit traffic lights are defined to control the straight-traveling, left-turning, and right-turning traffic flows at the exit at the same time. According to the upstream and downstream signal light states, the change of the overflow index of the short-distance road section can be described as follows.

**Fig 3:**
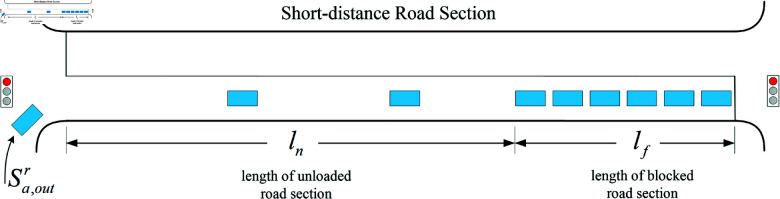
The traffic flow distribution diagram when traffic lights in both entrance and exit of the short-distance road section are red.

Case 1: When the traffic light at the entrance of the short-distance road section is red and the exit is also red, at this time, the target road section has only right-turning vehicles from the upstream intersection, and the process is schematized as Fig 3.

The predicted values of the overflow index at this time are as follows:


$$\begin{array}{l} OP^{\prime }=\frac{l_{n}-l_{c}^{\prime }}{L} \end{array}$$ (8)

$$\begin{array}{l} BP^{\prime }=\frac{l_{f}+l_{c}^{\prime }}{L} \end{array}$$ (9)

where ${OP}^{\prime }$is the predicted value of the overflow parameter,$l_{c}^{\prime }$ is thelength of the road section occupied by the new queuing vehicles in the predicted timeperiod $T_{p}$.

When the signal light status of the intersection upstream of the target road section isred, the change of this value is mainly related to the convergence of right-turningvehicles, and the specific expression is as follows:


$$\begin{array}{l} l_{c}^{\prime }=v_{xt1}\cdot T_{p} \end{array}$$ (10)

where $v_{xt1}$ is the stop-wave speed of the right-turning traffic flow, and its relationship with the traffic flow rate $S_{a,\text{out}}^{r}$ of the right-turning traffic into the short-distance road section is as follows [25]:


$$\begin{array}{l} v_{xt1}={\left(\frac{{u_{f}}^{2}}{4}-\frac{u_{f}}{k_{j}}\cdot s_{a,\text{ out }}^{r}\right)}^{\frac{1}{2}}-\frac{u_{f}}{2} \end{array}$$ (11)

where $u_{f}$ is the free-flow speed and $k_{j}$ is the blockage density.

**Fig 4:**
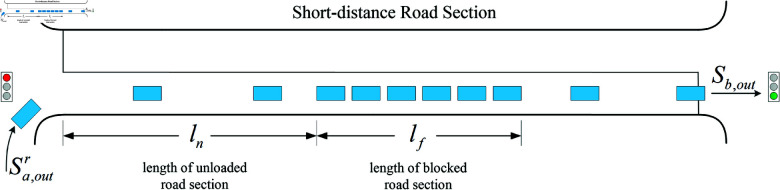
The traffic flow distribution diagram when the traffic light in entrance of the short-distance road section is red while the traffic light in exit of the short-distance road section is green.

Case 2: When the traffic light at the entrance of the short-distance road section is red and the exit is green, at this time, the change of traffic flow in the target road section involves the right-turn entering traffic at the upstream intersection and exiting at the downstream intersection, and the process is schematically shown as Fig 4.

The predicted values of overflow index at this time are as follows:


$$\begin{array}{l} OP^{\prime }=\frac{l_{n}-l_{c}^{\prime }}{L} \end{array}$$ (12)

$$\begin{array}{l} BP^{\prime }=\frac{l_{f}+l_{c}^{\prime }-l_{g}}{L} \end{array}$$ (13)

where $l_{g}$ is the roadlength occupied by vehicles exiting from the downstream exit of the road section in the predictiontime period $T_{p}$.

When the signal light status downstream of the target road section is green, thechange of this value is mainly related to the flow rate of the exiting vehicles; thespecific expression is as follows:


$$\begin{array}{l} l_{g}=v_{q}\cdot T_{p} \end{array}$$ (14)

where $v_{q}$ is the start-wave speed, and when $l_{f}$ decreases to a critical threshold, ${BP}^{\prime }$ decreases to 0 and does not continue to decrease. The specific expression is as follows:


$$\begin{array}{l} BP^{\prime }=0,l_{f}\leq \left(S_{b,\text{out}}-S_{a,\text{out}}^{r}\right)\cdot T_{p}\cdot h_{o} \end{array}$$ (15)

where $S_{b,\text{out}}$ is the outflow rate for the short-distance road section, $h_{o}$ is the headspace of the vehicle.

**Fig 5:**
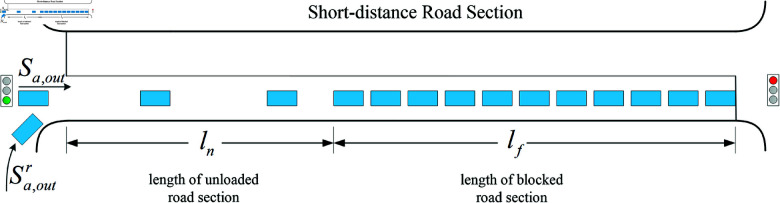
The traffic flow distribution diagram when the traffic light in entrance of the short-distance road section is green while the traffic light in exit of the short-distance road section is red.

Case 3: When the entrance traffic light of the short-distance road section is green and the exit is red, at this time, the change of traffic flow on the target road section is mainly related to the left-turn, straight and right-turn traffic at the upstream intersection, and the process is schematically shown as Fig 5.

The predicted values of overflow index at this time are as follows:


$$\begin{array}{l} OP^{\prime }=\frac{l_{n}-l_{c}^{\prime }}{L},BP^{\prime }=\frac{l_{f}+l_{c}^{\prime }}{L} \end{array}$$ (16)

In the Eq (16), the change of $l_{c}^{\prime }$ is mainly related to the phase of the green light and the exiting flow rate in the right-turn direction, and the specific expression is as follows:


$$\begin{array}{l} l_{c}^{\prime }=v_{xt2}\cdot T_{p} \end{array}$$ (17)

where $v_{xt2}$ is the stop-wave speed of right-turning and non-right-turning traffic approaching, and its relationship with the traffic flow rates $s_{a,\text{out}}^{r}$ and $S_{a,\text{out}}$ entering the short-distance road section is as follows:


$$\begin{array}{l} v_{xt2}={\left(\frac{{u_{f}}^{2}}{4}-\frac{u_{f}}{k_{j}}\cdot \left(s_{a,\text{out}}^{r}+s_{a,\text{out}}\right)\right)}^{\frac{1}{2}}-\frac{u_{f}}{2} \end{array}$$ (18)

$$\begin{array}{l} S_{a,\text{out}}=S_{a,\text{out}}^{\text{str}}+S_{a,\text{out}}^{\text{left}} \end{array}$$ (19)

where $S_{a,\text{out}}$is related to the actual queued vehicles in the green light release phase, and the valueis reduced to the corresponding input flow rate when the queue is completelydissipated.

**Fig 6:**
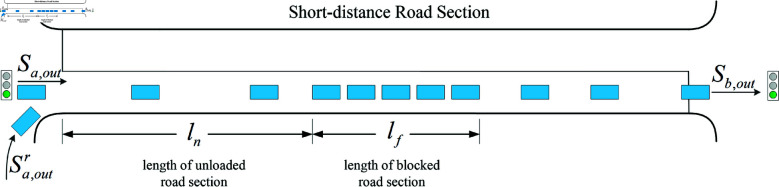
The traffic flow distribution diagram when traffic lights in both entrance and exit of the short-distance road section are green.

Case 4: When the entrance traffic light of the short-distance road section is green and the exit is also green, at this time, the change of traffic flow in the target road section is not only related to the upstream intersection’s straight, left-turning, and right-turning traffic, but also affected by the outgoing flow rate of the road section, and the process is schematized as Fig 6.

The predicted values of overflow index at this time are as follows:


$$\begin{array}{l} OP^{\prime }=\frac{l_{n}-l_{c}^{\prime }}{L},BP^{\prime }=\frac{l_{f}+l_{c}^{\prime }-l_{g}}{L} \end{array}$$ (20)

The specific expressions for $l_{c}^{\prime }$ and $l_{g}$ in the above equation are as follows:


$$\begin{array}{l} l_{c}^{\prime }=v_{xt2}\cdot T_{p} \end{array}$$ (21)

$$\begin{array}{l} l_{g}=v_{q}\cdot T_{p} \end{array}$$ (22)

### Active control model based on deep reinforcement learning

The key to active control of traffic signals at short-distance intersections is to take signal control actions that are compatible with the current real-time and predicted state of the system. This will generate the optimal control scheme, so that short-distance intersections can reduce the probability of overflow occurring in the target road section under the premise of ensuring the overall traffic efficiency.

**Fig 7:**
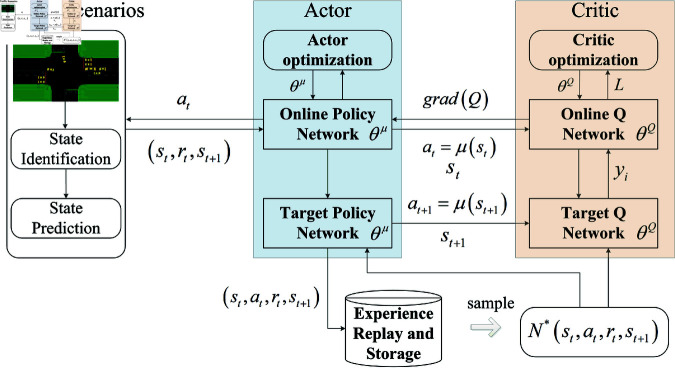
Diagram of the reinforcement learning architecture based on DDPG proposed in this paper.

Considering that the active control process needs to consider the real-time traffic demand of the target road section and the trend of the overflow indicator, and generate the signal control scheme for two intersections, and also realize the dual objectives of traffic efficiency improvement and overflow control. For this multi-input and multi-output control demand, this paper adopts the Deep Deterministic Policy Gradient (DDPG) algorithm to solve the control problem in continuous state space and continuous action space (Fig 7).

The DDPG algorithm used in this paper employs target networks suitable for Actor-Critic, and instead of directly copying the network weights for parameter updating, uses “soft” target updating by creating copies $Q^{\prime }\left(s,a|\theta^{Q^{\prime }}\right)$ and $\mu^{\prime }\left(s|\theta^{\mu^{\prime }}\right)$ of the Actor and Critic networks, respectively, to be used to compute the target values. The weights of these target networks are then updated by allowing them to slowly track the learned network, which means that the target values are constrained to change slowly, thus greatly improving the stability of the learning.

#### State.

The primary control objective of short-distance intersections is the overall traffic efficiency of the target scenario, that is, the response to traffic demand. Therefore, the real-time traffic demand in each direction of the upstream and downstream intersections is the base state. In this paper, the number of vehicles in each direction $q_{i}^{A}$ is used as one of the state vectors of the control system, that is, the number of vehicles existing in the direction *i* of intersection *A*. Overflow control for short-distance road sections is another key control goal, so in this paper, the current value  (OP,BP) and the predicted value $\left(OP^{\prime },BP^{\prime }\right)$ of the overflow index are selected as one of the state vectors.

In the traffic signal control system, considering the traffic safety factor, the generation of signal control action needs to satisfy a certain time limit (maximum green light time, minimum green light time), so here the current signal phase $ph_{i}$ with road right-of-way and green light duration $T_{phi}^{A}$ are taken as one of the elements of the state vector. When the number of directions at the upstream and downstream intersections are *M* and *N* respectively, the corresponding number of vehicles are $\left[q_{1}^{A},q_{2}^{A},\ldots ,q_{M}^{A}\right]$ and $\left[q_{1}^{B},q_{2}^{B},\ldots ,q_{N}^{B}\right]$. Therefore, the state vector can be constructed as follows:


$$\begin{array}{l} S={\left[q_{1}^{A},\cdots \;,q_{M}^{A},q_{1}^{B},\cdots \;,q_{N}^{B},OP,BP,OP^{\prime },BP^{\prime },ph_{1},\cdots \;,ph_{i},T_{ph1},\cdots \;,T_{phi}\right]}^{T} \end{array}$$ (23)

#### Action.

The design of the action space depends to some extent on the roadway base channelization and the corresponding traffic demand. Considering that the scenarios studied in this paper contain short-distance road sections, single-direction release (simultaneous release of left-turn and straight traffic) can cope with the risk of overflow more efficiently. Meanwhile, considering the adaptability of signal coordination at upstream and downstream intersections, the design of signal control actions at upstream and downstream intersections in this paper adopts the form of single-direction release, and the sequence of release is not fixed and does not show periodicity.

**Fig 8:**
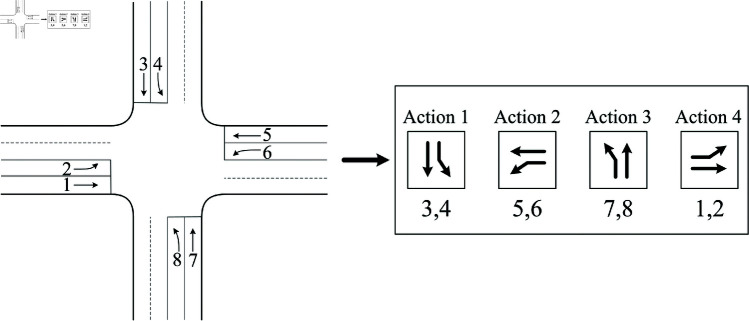
Action space schematic based on the distribution of traffic flow in each direction at the intersection.

The action space studied in this paper is a set of signaling control actions that act on two intersections simultaneously. The action space is defined here as $A=\left\{a_{i},b_{j}\right\}$, where $a_{i}$ is the action at intersection *a* and $b_{i}$ is the action at intersection *b*. Fig 8 shows the relationship between action and phase. The switching of actions firstly involves the problem of maximum and minimum time limits of green lights. In this paper, we adopt the action feedback treatment, where a larger penalty value will be assigned to the action for the green light duration exceeding the range $\left[g_{\min},g_{\max}\right]$. This value is directly added to the *Q* value corresponding to the action, which in turn makes the *Q* value of the action behavior in the action space decrease and not be selected, ensuring that the green light duration of the signaling action is within a reasonable range. Compared with limiting the green light duration in the reward function, this kind of feedback processing of internal action values at the early stage of action selection can simplify the learning process and improve the learning efficiency.

#### Reward.

The reward function is a comprehensive evaluation of the generated action space and affects the next signaling decision process. Consider that travelers expect to spend a sufficiently short time and make a sufficiently low number of stops when passing through short-distance intersections. On the other hand, managers expect short-distance intersections to be as free from overflow problems as possible. The design of the reward function in this section emphasizes three main aspects of performance, which are the overall stopping delay of the short-distance scenario, the overflow state of the short-distance road section, and the number of stops within the short-distance road section.

Conventional overflow indexes can only evaluate the signal control action in two states, namely, overflow has occurred and overflow has not occurred, which corresponds to a sparse distribution of reward function values and makes it difficult for the intelligent body to explore the final goal. In this paper, we design the overflow index and its predicted values so that the overflow status of short-distance road sections can be represented in a linear and continuous manner. This greatly refines the process of signal control actions on the overflow state, making the intelligent body’s exploration of the optimization scheme more active and efficient.

The reward function metrics proposed in this paper are specified as follows:

1) The cumulative stopping delay difference $r_{\text{delay}}$ between neighboring decision points. If we are currently at decision time point *t* + 1, the cumulative vehicle stopping delay difference at this point is $r_{\text{delay}}=w_{t+1}-w_{t}$, $w_{t}$ is the total stopping delay at the corresponding moment. If the value $r_{\text{delay}}>0$,it indicates that the action is not conducive to the overall traffic efficiency.

2) The overflow state of the target road section $r_{\text{overflow}}$. If we are currently at decision time point *t* + 1, when the predicted value of the overflow index is $\left(OP^{\prime },BP^{\prime }\right)$, the overflow state indicator is designed in the form of a positive correlation with the value of $OP^{\prime }$ and a negative correlation with the value of $BP^{\prime }$, that is, $r_{\text{overflow }}=k_{o}\cdot OP^{\prime }+k_{b}\cdot \left(1-BP^{\prime }\right)$. This reward function indicates that the smaller the ratio of the length of the remaining road section, or the longer the blocking convoy, the higher the probability of overflow occurrence, and the lower the reward value of the corresponding overflow state.

3) Number of vehicles taking stopping maneuvers within the target roadway ($r_{\text{stopNum}}$).

Combining the above factors with the corresponding weighting coefficients $k_{1}$,$k_{2}$,$k_{3}$, the final reward function is as follows:


$$\begin{array}{l} r=k_{1}\cdot r_{\text{delay}}+k_{2}\cdot r_{\text{overflow}}+k_{3}\cdot r_{\text{stopNum}} \end{array}$$ (24)

## Experiments

### Parameter settings for simulation experiments

**Fig 9:**
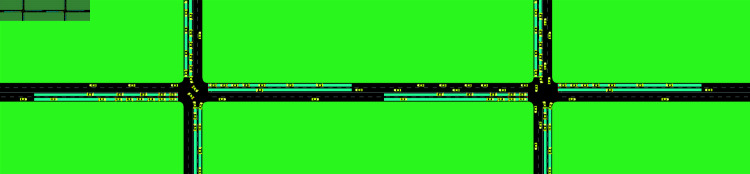
Traffic simulation of the intersections.

In order to test the short-distance intersections active control method proposed in this paper, this paper builds a simulation environment of two intersections with reference to urban intersections with a spacing of 245 meters (Fig 9), and each road section of the intersections has 4 lanes in both directions. The traffic flow data of each road section is shown in Table 1.

**Table 1:** The traffic flow data of experimental intersections.

		Intersection A	Intersection B
**Time period**	Direction	Traffic flow ( *veh* ∕ *h* )	Traffic flow ( *veh* ∕ *h* )
**Flat peak** **time period**	North	660	750
	East	570	600
	South	780	740
	West	550	550
**Peak time** **period**	North	1100	1250
	East	950	1000
	South	1300	1240
	West	910	920

The eastbound road section at intersection A and the westbound road section at intersection B are short-distance road section.

This paper builds a simulation experimental environment based on the microscopic traffic simulation software SUMO, and uses the Traci interface provided by SUMO to send the state information to the signal controller built under the Python environment to realize the simulation of the active optimization control process of traffic signals.

Considering that traffic signal control is to be performed for two intersections, in the training process of the learning algorithm, the moment when the key traffic flow obtains the right-of-way is used as the training reference point, where the key traffic flow is selected from the short-distance road section. In this paper, the signal control process between the three training reference points is a training set, and the deep reinforcement learning model conducts a total of 350 training sets. The relevant parameters of the deep reinforcement learning model are set as follows: the discount factor *γ* is set to 0.95, the learning rate *α* of the Actor network is 0.0001, the learning rate *β* of the Critic network is 0.001, the hidden layer in the neural network adopts three fully-connected networks, the sampling parameters *N* of the learning process is 128, the pool of the empirical playback *M* is 100,000, and the parameter of the soft updating rate *τ* is 0.001. The reward function of the overflow index parameters $k_{o},k_{b}$ are 6 and 4, respectively, and the weight of each parameter in the reward function is set to $k_{1}=0.65,k_{2}=0.05,k_{3}=1$, so that the three reward indexes $r_{\text{stopTime}},r_{\text{overflow}},r_{\text{stopNum}}$ satisfy the normalized quantitative requirements.

### Experimental results and analysis

#### Validation of the overall control effectiveness of the method.

In order to investigate the control effect of the short-distance traffic scenario signal control method based on overflow prediction proposed in this paper, the effect of this method (P_DDPG) in terms of overflow suppression is firstly simulated and tested. In order to make a comparative evaluation of the control effect, the method without overflow index prediction data (DDPG) under the same condition is selected here for comparison, and the green wave coordinated control method [13] (CTC) with phase offset coordination technique in the short-distance scenario is also selected as a comparison.

**Fig 10:**
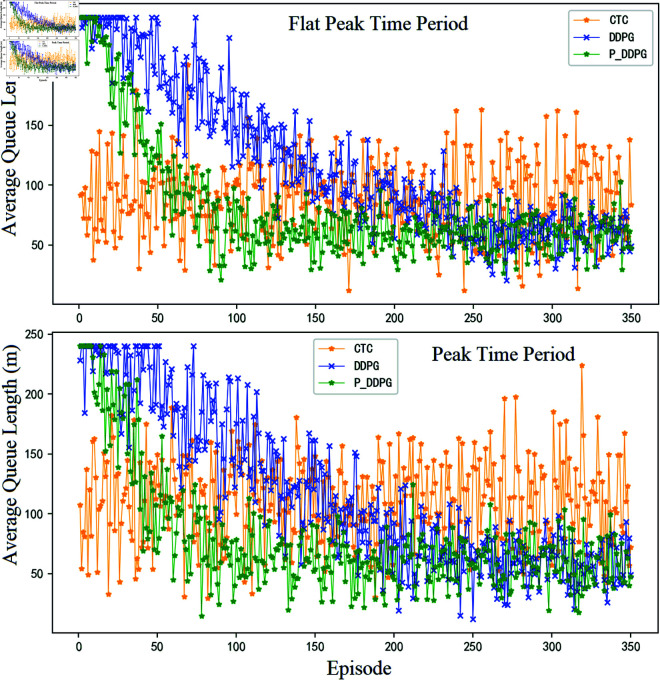
Comparison of the methods proposed in this paper in terms of average queue length metrics at intersections for different time periods.

For the selection of overflow suppression metrics, the average queue length metric within each training cycle of a short-distance road section is used here. Where the average queue length index is obtained by accumulating the value of the maximum queue in the target direction within s signal cycles contained in a single training set, and then dividing it by the number of cycles s in the training set. For the overflow phenomenon that occurs in the simulation experiment, the experiment is ensured here by emergency switching of the phase of the converging traffic in the overflow section. Fig 10 shows the specific performance of the control process, in which the flat peak time period and peak time period are selected as 10:30-11:30 and 17:30-18:30, respectively, and the flow data is the average flow of the corresponding time period.

**Fig 11:**
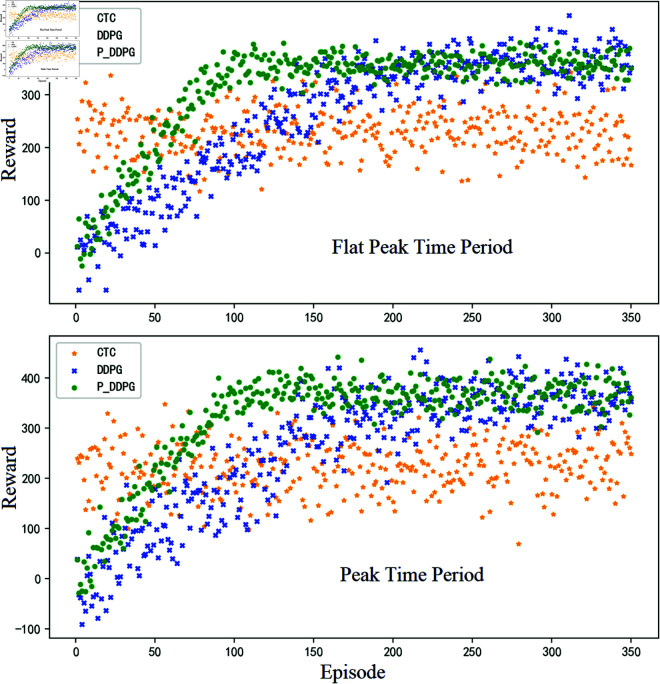
Comparison of the methods proposed in this paper in terms of reward value distribution at intersections for different time periods.

The control goal of the short-distance traffic scenario is not only the control of queue length within the short-distance road section, thus realizing the overflow prevention and control goal of the critical road section. It is also necessary to ensure the overall traffic efficiency on this basis. For the selection of the overall control effect index of the short-distance scenario, the overall reward value of the two intersections is used here, that is, the total reward value in each training cycle, and the specific experimental results are shown in Fig 11.

From the above experimental results, it can be seen that both reinforcement learning algorithms have a tendency to converge, indicating that both algorithms are suitable for traffic signal control problems. It can also be seen from the figure that when P_DDPG and DDPG learning algorithms finally converge, in terms of reducing the queue length of the target road section, P_DDPG and DDPG have less improvement than the conventional modeled CTC algorithm, but P_DDPG and DDPG are able to guarantee the overall efficiency of the passage of the two intersections at the same time, and the improvement in the overall efficiency is more obvious.

It can be calculated that the active control method P_DDPG method and the conventional DDPG method, compared with the conventional coordinated control method CTC, can reduce the queue length by 8.4% and 6.7% respectively (the reduction ratio of the mean value of the average queue length indicator in the converged state), and the overall reward gain is improved by 20.6% and 18.9% respectively (the improvement ratio of the mean value of the cumulative reward in the converged state) . It can also be seen that the larger the traffic volume and the more complex the control process in a traffic scenario, the conventional modeled approach will gradually reveal its corresponding shortcomings, while the model-less learning algorithm will have a certain advantage.

**Fig 12:**
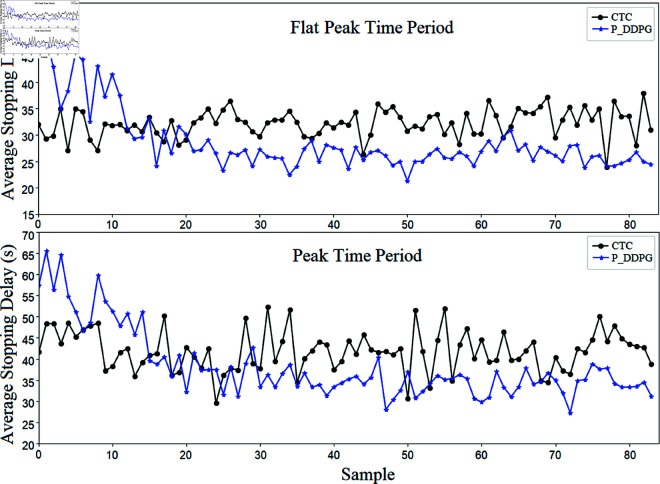
Comparison of the methods proposed in this paper in terms of average stopping delay at intersections for different time periods.

The above simulation results validate and analyze the method proposed in this paper in terms of both short-distance road section queue length and total reward value. In order to further confirm the connection between the improvement of the total reward value and the actual control effect of the intersection, the average stopping delay indexes of vehicles passing through this traffic scenario are counted and analyzed here. In this paper, by slicing and dicing the continuous dataset in the experiment and averaging the average vehicle delays in each sub-dataset (each sub-dataset contains four base datasets), the statistical results as shown in Fig 12 are obtained.

As can be seen from the experimental results graph, the present algorithm is not only able to realize the improvement of the overall reward value, but also has the same obvious effect in the regular operation evaluation index. The active control method proposed in this paper is able to reduce the average stopping delay of vehicles while also making the fluctuation of indicators during intersection operation significantly lower. Especially when the traffic flow saturation is relatively high, this method is able to take active optimization measures according to the trend of the state change of the key road sections to avoid the problem of road section overflow that is prone to occur when the traffic flow is high.

By counting the experimental data, the degree of improvement of the P_DDPG method relative to the CTC method when it reaches the converged state can be calculated (as shown in Table 2).

**Table 2:** Effectiveness improvement of the P_DDPG method proposed in this paper compared to the traditional CTC method.

	Reward	Average stopping delay
**Flat peak time period**	17.658%	13.149%
**Peak time period**	19.114%	14.534%

From the statistical results in the table above, it can be seen that the method proposed in this paper has an obvious effect on the improvement of the overall operation effect of the intersection. Combining the control process of the two learning algorithms can also show that the learning algorithm with overflow prediction proposed in this paper is able to reach the convergence state faster. From the cumulative reward graph, it can be seen that compared with the DDPG algorithm without prediction function, the P_DDPG method is able to improve 14.2% and 15.6% (the improvement ratio of the mean value of the cumulative reward in the converged state) in the flat peak time period and peak time period, respectively, which can not only show that the method with overflow state prediction can explore the better state-action relationship faster, but also in the more complex the traffic scene, the more obvious the efficiency advantage of this learning exploration. The more obvious the efficiency advantage of this learning exploration.

#### Validation of the reward function.

Aiming at the reward sparsity problem of the overflow state in the conventional learning algorithm, this paper designs an overflow state feedback function. In order to verify the effect of this state feedback function, this paper conducts a comparison experiment on it. Here, the conventional feedback function is chosen as a control, that is, a larger penalty value is obtained for overflow once, and a constant reward value is obtained for no overflow.

**Fig 13:**
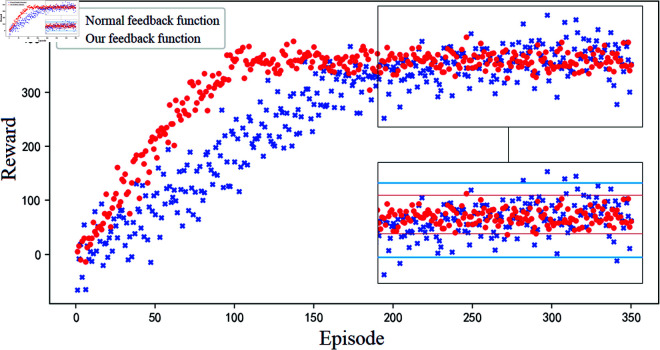
Comparative validation of overflow state feedback indicator in reward functions.

From the experimental results (Fig 13), it can be seen that after designing the corresponding refined feedback indicators for the overflow state, the convergence speed of the algorithm is significantly increased and the fluctuation of the action’s reward is significantly reduced. As can be seen by the change range of the cumulative reward value of the two methods before and after reaching the converged state, the feedback function proposed in this paper containing the refined overflow state index can reduce the exploration range by 21.7% and 18.5% before and after convergence respectively (the sample variance formula is used here to characterize the index), which also provides a more sensitive action exploration feedback for the algorithm’s learning process, and can better guide the algorithm to find the optimal solution and make the control strategy more proactive.

In this paper, when designing the reward function, we refer to the factors affecting the accessibility of short-distance traffic scenarios, and add an indicator of Times Indicator of Stopping (TIS) on the target road section. This indicator is to promote the learning algorithm to be able to tend to realize the effect of green wave coordination at upstream and downstream intersections. The effect of this indicator is analyzed here through the specific performance of the learning process before and after blocking this indicator.

**Fig 14:**
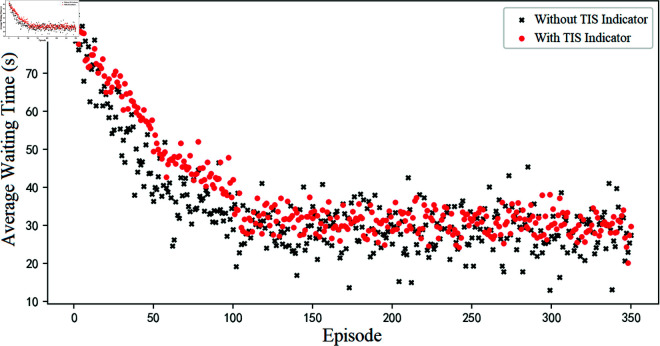
Comparative validation of TIS indicator in reward functions.

From the comparison results (Fig 14), it can be seen that the indicator of TIS reduces the rate of convergence, but the fluctuation of the average waiting time is reduced by 12.6% (the sample variance formula is used here to characterize this indicator). This suggests that as the average times of vehicle stops within a short-distance road section decrease, the capacity level of the short-distance road section becomes relatively stable, and this stable traffic environment can greatly reduce the risk of overflow occurring.

## Conclusion

This paper introduces an active control method for short-distance intersections based on overflow state prediction, which improves the hysteresis and complexity of signal control schemes by capturing the operational state of traffic flow on road sections. In this paper, an overflow index prediction model and a deep reinforcement learning model are successively designed to improve the proactivity and integration capability of the signaling scheme. It is demonstrated through experiments that the control method considering overflow prediction can effectively reduce the overflow risk of short-distance intersections, ensure the overall access efficiency, and reduce the access delay.

## Supporting information

S1 Table Intersection information in the simulation environment. (XLSX)

S2 Table Parameter description. (XLSX)

S3 PDF The learning process of deep reinforcement learning algorithms in this paper. (PDF)

S4 RAR Data corresponding to the values of the figures and indicators. (ZIP)
