# Supplementary material for: Traffic signal active control method for short-distance intersections
Source: PLoS One. 2025 Mar 14;20(3):e0319804. doi: 10.1371/journal.pone.0319804 (PMC11908704; doi:10.1371/journal.pone.0319804)
Supplement: S3 PDF — (PDF) [file pone.0319804.s003.pdf]

The learning process of the P_DDPG algorithm is as follows:

Inputs: reward discount factor γ, Actor network learning rate α, Critic network learning rate β, soft update rate parameter τ to the target network, sampling pool parameter N and experience playback pool M;

Initialize the weights θ^{μ} and θ^{μ} of the Actor and Critic networks;

Initialize the weights $\theta^{Q'} \leftarrow \theta^{Q}, \theta^{\mu'} \leftarrow \theta^{\mu}$ of the target networks Q' and μ';

Initialize the experience playback cache D

Begin

1: **for** episode=1,2,⋯,M do

2: Sampling a random noise from the noise process $\mathcal{N}$ and adding it to the behavioral strategy to prepare for the exploration of implementing intersection signal control actions

3: Receive data values s_1 of queuing vehicles in each direction in the scene, overflow status of the target roadway, etc.

4: for t=1,2,⋯,T do

5: Based on the current policy and noise, from the set of signaling actions, select the signaling control action $a_t = \mu(s_t|\theta^{\mu}) + \mathcal{N}_t$

6: Execute the signaling action a_t and receive the reward r_t consisting of the evaluation indexes of both the overall operational effectiveness of the intersection and the overflow control effectiveness of the short connecting section and the new intersection state value s_{t+1}

7: Update the sample pool D, construct the associated data into sample units $\{s_t, a_t, r_t, s_{t+1}\}$ and store them, and complete the replacement of the old and new update of the empirical information consisting of the state of each part of the intersection, the signal control action, and the intersection operation revenue.

8: A random sample of the smallest batch size from D with a cache range of N is used to compute and form the signal control policy

9: Calculate the objective value of the value function

$$y_i = R_{i+1} + \gamma \cdot Q'\left(S_{t+1}, \mu'\left(S_{i+1}|\theta^{\mu'}\right)|\theta^{Q'}\right)$$

10: Update the Critic network with the minimization loss L_l, that is

$$L_l = \sum_i \left(y_i - Q(S_i, A_i|\theta^{Q})\right)^2 / n$$

11: Update the Actor network with sampling gradient, that is

$$\nabla_{\theta\mu}\mathcal{J} \approx [\sum_i \nabla_a Q(s, a|\theta^{Q})|s = s_i, a = \mu(s_i)\nabla_{\theta^{\mu}} \mu(s|\theta^{\mu})|s_i]/N$$

12: Update the target network: $\theta^{Q'} \leftarrow \tau\theta^{Q} + (1-\tau)\theta^{Q'}$, $\theta^{\mu'} \leftarrow \tau\theta^{\mu} +$

$(1-\tau)\theta^{\mu'}$, and step by step determine the correlation of states, actions and reward values in the signal control strategy.

13: **end for**

14: **end for**

end.
